# The rise of point-of-care ultrasound in cardiopulmonary diagnostics

**DOI:** 10.1093/ehjimp/qyaf147

**Published:** 2026-01-01

**Authors:** Marina Petersen Saadi, Guilherme Heiden Telo, Prayuth Rasmeehirun, Erwan Donal

**Affiliations:** Department of Cardiology, Hospital de Clínicas de Porto Alegre, Ramiro Barcelos 2350, 90035-003 Porto Alegre/RS, Brazil; Department of Cardiology, University of Rennes, Pontchaillou Hospital - CHU Rennes, Inserm, LTSI—UMR 1099, Rennes F-35000, France; Department of Cardiology, Hospital de Clínicas de Porto Alegre, Ramiro Barcelos 2350, 90035-003 Porto Alegre/RS, Brazil; Department of Cardiology, University of Rennes, Pontchaillou Hospital - CHU Rennes, Inserm, LTSI—UMR 1099, Rennes F-35000, France; Department of Cardiology, University of Rennes, Pontchaillou Hospital - CHU Rennes, Inserm, LTSI—UMR 1099, Rennes F-35000, France

**Keywords:** Point-of-Care Ultrasound, POCUS, heart failure, STEMI, cardiogenic shock

## Abstract

Point-of-care ultrasound (POCUS) has rapidly evolved from a diagnostic adjunct into an essential extension of bedside clinical reasoning in acute cardiovascular care. By providing immediate, physiologically grounded, and non-invasive information, POCUS enhances diagnostic accuracy, risk stratification, and therapeutic guidance in real time. Among its core applications, lung ultrasound enables reliable detection and monitoring of pulmonary congestion, outperforming traditional methods such as chest X-ray and physical examination. The Venous Excess Ultrasound Score offers a structured assessment of systemic venous congestion through abdominal venous Doppler patterns. The left ventricular outflow tract velocity–time integral serves as a reproducible surrogate of forward flow and cardiac output, while focused cardiac ultrasound provides rapid structural and functional evaluation of the heart. The reliability and prognostic value of these modalities have been supported by growing evidence across diverse clinical contexts, though standardization of training and acquisition protocols remains crucial for widespread implementation. Integration of POCUS into daily workflows—through structured, serial assessments of pulmonary, venous, and haemodynamic status—holds promise to refine decision-making, individualize treatment strategies, and improve outcomes. This review summarizes current evidence, methodological considerations, and practical implications of POCUS in acute cardiovascular medicine, emphasizing its complementarity to, rather than replacement of, traditional diagnostic tools.

## Graphical Abstract

**Graphical Abstract:**
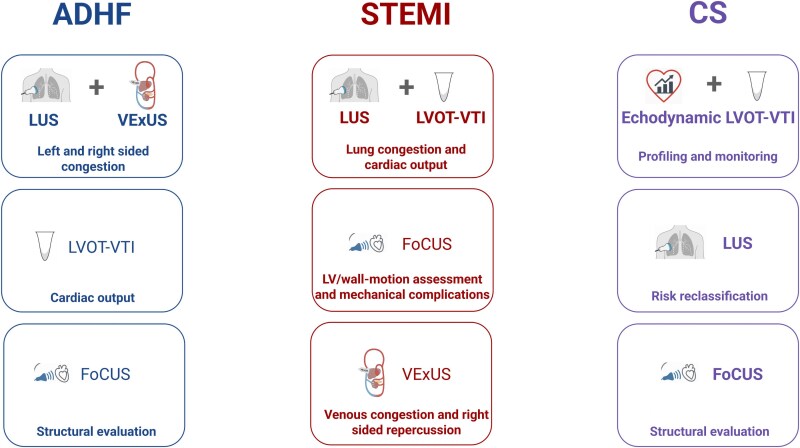
Point-of-care ultrasound (POCUS) provides condition-specific multimodal assessment in acute cardiovascular care. In acute decompensated heart failure, it integrates lung ultrasound (LUS) and Venous Excess Ultrasound Score (VExUS) for congestion profiling, complemented by left ventricular outflow tract velocity–time integral (LVOT-VTI) for flow evaluation and focused cardiac ultrasound (FoCUS) for structural assessment. In ST-elevation myocardial infarction, LUS and LVOT-VTI assess pulmonary congestion and forward flow, FoCUS identifies ventricular dysfunction and mechanical complications, and VExUS evaluates systemic venous repercussion. In cardiogenic shock, echodynamic profiling with LVOT-VTI and related parameters supports serial haemodynamic monitoring, while LUS aids risk stratification and FoCUS enables structural and functional evaluation.

## Introduction

Accurate and timely cardiopulmonary diagnosis is a key part of cardiovascular care. However, traditional bedside tools often lack the sensitivity and immediacy required for optimal decision-making. Physical examination, though indispensable, is limited by interobserver variability and low accuracy—pulmonary rales detect right atrial pressure (RAP) ≥10 mmHg and left atrial pressure ≥20 mmHg in only 28% and 25% of cases, respectively, while jugular venous distension shows a sensitivity of just 39% for systemic congestion.^1^ Chest radiography performs only modestly better, with a sensitivity of ∼56% for detecting pulmonary congestion in acute decompensated heart failure (ADHF).^2^ Although biomarkers, imaging, and invasive haemodynamic monitoring remain valuable, they are either delayed, non-specific, or not readily available at the bedside. These limitations underscore the need for point-of-care ultrasound (POCUS), which offers real-time, non-invasive, and physiologically grounded assessment directly integrated into clinical decision-making.

POCUS refers to a focused, goal-oriented ultrasound examination performed by the clinician at the bedside and integrated into clinical reasoning. By providing real-time insights, POCUS complements—rather than replaces—the physical examination and conventional diagnostic tools. It enhances diagnostic accuracy, supports risk stratification, and guides therapy, expediting clinical decision-making across diverse clinical environments.

In cardiovascular medicine, lung ultrasound (LUS) identifies pulmonary congestion through B-lines with markedly higher accuracy than traditional methods.^3^ The Venous Excess Ultrasound Score (VExUS) quantifies systemic venous congestion through abdominal venous Doppler patterns, reflecting venous afterload and the downstream transmission of pressure that contributes to end-organ injury.^4^ Focused cardiac ultrasound (FoCUS) represents a specific type of POCUS applied to the heart—performed by clinicians appropriately trained in its use, though not necessarily in comprehensive echocardiography. It serves as an extension of the physical examination, enabling rapid assessment of global left and right ventricular function, structural alterations, and detection of pericardial effusion. Echodynamic assessment refers to the non-invasive evaluation of haemodynamics using bedside echocardiography, combining Doppler- and morphology-based parameters to estimate cardiac output, filling pressures, and vascular resistance. Together, these modalities broaden bedside haemodynamic evaluation, integrating clinical and imaging data to refine diagnostic reasoning and guide therapy.^5^

This narrative review explores the evolving role of POCUS as a comprehensive, physiology-based tool for cardiopulmonary diagnostics and acute cardiovascular care. Beyond individual applications, such as LUS for pulmonary congestion or the VExUS for systemic venous overload, the review integrates additional haemodynamic modalities—including the left ventricular outflow tract velocity–time integral (LVOT-VTI) and FoCUS to illustrate how multimodal POCUS can provide real-time insights into both congestion and perfusion. Together, these approaches are redefining bedside haemodynamic assessment and clinical decision-making across ADHF, ST-elevation myocardial infarction (STEMI), and cardiogenic shock (CS), where rapid and accurate evaluation is essential.

## Acquisition and interpretation

### Lung ultrasound

**Figure 1:**
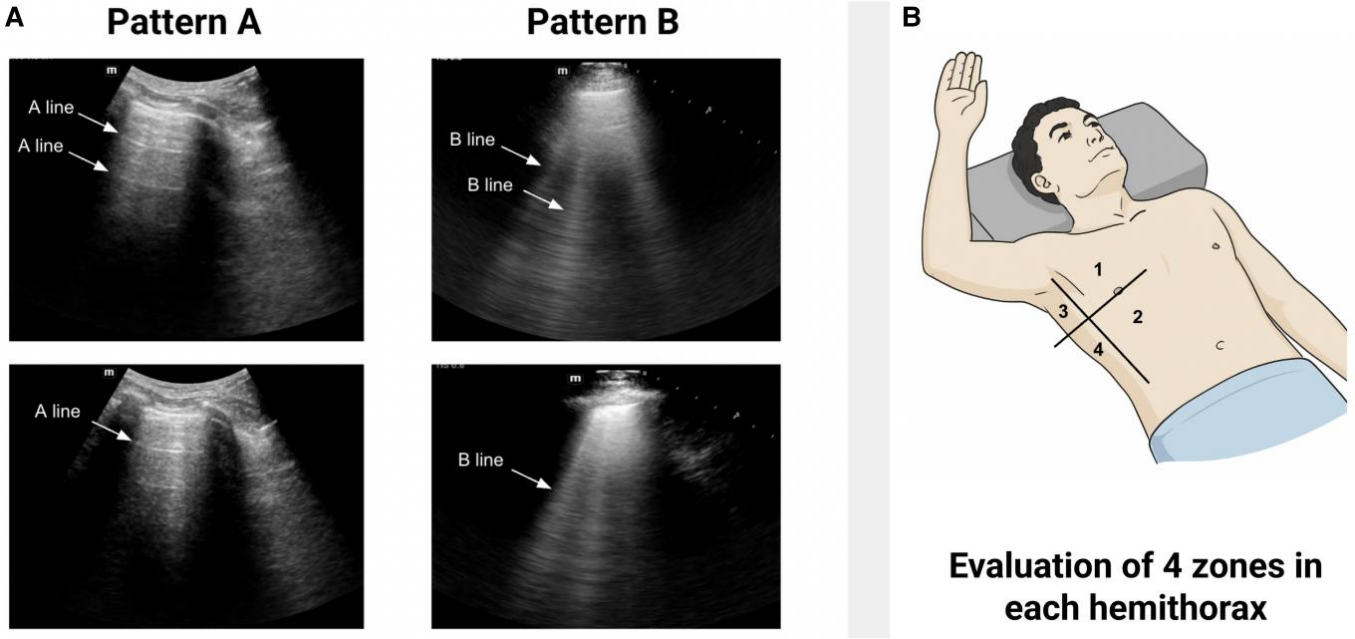
Lung ultrasound patterns and scanning protocol. (*A*) Pattern A shows normal lung aeration, with horizontal reverberation artefacts (A-lines) beneath a continuous pleural line. Pattern B shows interstitial syndrome, characterized by multiple vertical artefacts (B-lines) that arise from the pleura, reach the bottom of the screen, and erase A-lines. (*B*) The 8-zone lung ultrasound protocol involves the evaluation of four regions per hemithorax (two anterior and two lateral), as shown, with the patient in a supine or semi-recumbent position.

LUS is based on the interpretation of artefacts rather than direct visualization of anatomical structures. In normally aerated lungs, the only visible structure is the pleura, which appears as a hyperechoic horizontal line—known as the pleural line—that moves synchronously with respiration. Below it, horizontal reverberation artefacts called A-lines indicate preserved air content. As aeration decreases and interstitial fluid accumulates, vertical artefacts known as B-lines emerge. These laser-like, hyperechoic lines arise from the pleura, reach the bottom of the screen, move with breathing, and erase A-lines, as illustrated in *Figure 1A*.^6^

While B-lines are a hallmark of pulmonary edema in ADHF, they are not disease-specific and may also appear in other conditions that reduce lung aeration. Diffuse, non-gradient patterns may also occur in parenchymal lung diseases—such as interstitial lung disease, pulmonary fibrosis, or acute respiratory distress syndrome—whereas a basal-to-apical gradient of B-lines is consistent with cardiogenic congestion.^7^ Focal or asymmetric distributions usually indicate consolidation or atelectasis. Technical and patient-related factors can also modify artefact appearance: in obesity, excessive chest wall thickness attenuates ultrasound transmission, reducing B-line visibility, whereas in mechanically ventilated patients, increased lung volume and positive end-expiratory pressure PEEP can transiently suppress or redistribute B-lines. Accurate interpretation therefore requires integration with the clinical and haemodynamic context to avoid misclassification.^8^

Image acquisition is performed using a phased-array or convex probe, positioned perpendicular to the intercostal spaces to avoid rib shadowing, with depth adjusted to centre the pleural line. Among the available protocols, the 8-zone method—endorsed by the EACVI—offers an optimal balance between diagnostic accuracy and bedside feasibility, as illustrated in *Figure 1B*.^8^

### Venous Excess Ultrasound Score

The VExUS score is a composite Doppler-based tool designed to evaluate systemic venous congestion from the perspective of end-organ impact, integrating inferior vena cava (IVC) diameter with venous flow patterns in the hepatic vein (HV), portal vein (PV), and intrarenal veins (RV) to provide a physiologically grounded assessment.^9,10^

VExUS captures the haemodynamic consequences of elevated RAP, serving as an indirect marker of venous afterload. It reflects the balance between mean systemic filling pressure (MSFP) and RAP, which determines upstream venous pressure and modulates pressure transmission to abdominal organs. This backward transmission results in characteristic Doppler waveform abnormalities that correlate with increased risk of end-organ injury due to impaired capillary perfusion and interstitial edema.^11^

VExUS assessment begins with measurement of the IVC diameter 1–2 cm from the right atrium, with the patient in a supine position. If the IVC is dilated (≥2 cm), Doppler waveforms are obtained from the HV, PV, and RV using a phased-array or convex probe, either via a subcostal or transhepatic window. These waveforms are then used to grade the severity of venous congestion from 0 to 3, based on the degree of abnormal pressure transmission.

- **VExUS 0**: IVC < 2 cm—no significant venous congestion.
- **VExUS 1 (Mild)**: IVC ≥2 cm with either normal venous flow patterns or mildly abnormal findings [S < D in HV, PV pulsatility index (PVPI) 30–50%, or pulsatile but biphasic intrarenal flow].
- **VExUS 2 (Moderate)**: IVC ≥2 cm with at least one severely abnormal pattern (reversed S-wave in HV, PVPI ≥50% in PV, or monophasic intrarenal venous flow).
- **VExUS 3 (Severe)**: IVC ≥2 cm with two or more severely abnormal flow patterns.

**Figure 2:**
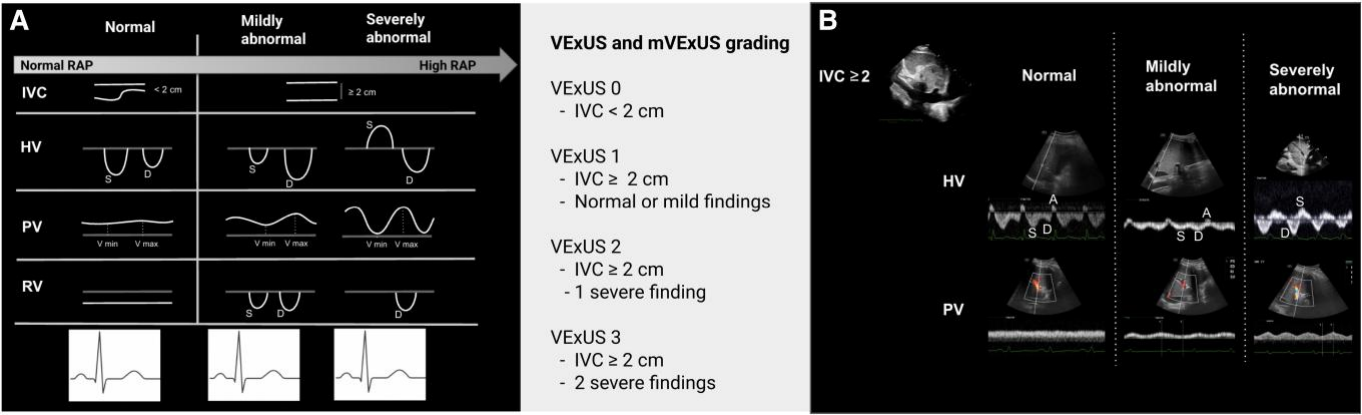
VExUS grading system and modified protocol. (*A*) Visual summary of the four VExUS grades, integrating IVC diameter and venous Doppler patterns from the HV, PV, and RV to stratify the severity of systemic venous congestion. (*B*) Modified VExUS (mVExUS) protocol, which excludes the renal vein. Flow patterns from HV and PV are categorized as normal, mildly abnormal, or severely abnormal to determine the final grade.

This stepwise evaluation offers a dynamic and organ-focused insight into the severity of venous congestion, expanding the haemodynamic assessment beyond IVC diameter alone, as detailed in *Figure 2A*. More recently, this approach has been simplified into the modified VExUS (mVExUS) protocol, by including only the assessment of the IVC, HV, and PV, excluding the renal vein—which is more time-consuming and operator-dependent—while maintaining diagnostic accuracy with a streamlined protocol as shown in *Figure 2B*.^12,13^ The mVExUS has demonstrated strong prognostic value in patients with ADHF, predicting in-hospital mortality^14^ with excellent interobserver reproducibility (intraclass correlation coefficient = 0.957) and a mean acquisition time of <5 min after a minimum of 50 supervised examinations, establishing it as a validated method in this setting.^15^

While VExUS is a valuable tool, its interpretation demands caution due to known pitfalls. High arrhythmic burden can complicate assessment, and transient changes like extrasystoles may be mistaken for dominant patterns. In cirrhosis and young people, PV pulsatility often reflects local hepatic changes rather than systemic congestion. A chronic S < D pattern may also occur in structural tricuspid regurgitation or pulmonary hypertension, irrespective of volume status. ECG correlation—particularly for HV interpretation—is essential to avoid misclassification.^16^ Clinical context is therefore fundamental.

### Left ventricular outflow tract velocity–time integral

LVOT-VTI is obtained using pulsed-wave Doppler in the apical five-chamber view, capturing the spectral envelope of flow across the LVOT. When indexed to the LVOT area, it enables estimation of stroke volume and cardiac output, although VTI alone is frequently used as a practical bedside surrogate of forward flow. Normal values range from 18 to 22 cm,^17^ while values <16 cm suggest impaired forward flow and have been associated with higher mortality in critically ill patients.^18^

Beyond its haemodynamic utility, LVOT-VTI has emerged as an independent prognostic marker across various settings. In STEMI, a cutoff of 14 cm identifies patients at greater risk of in-hospital death and early CS.^19^ In chronic heart failure with reduced ejection fraction, LVOT-VTI <15 cm independently predicted 28-month mortality with superior accuracy to left ventricular ejection fraction itself.^20^ In contrast, an optimal in-hospital prognostic cutoff has not yet been defined for ADHF. In venoarterial extracorporeal membrane oxygenation (VA-ECMO) support, a threshold near 10 cm has been proposed to guide weaning, with higher values indicating sufficient native output for decannulation.^17^

Accurate measurement requires meticulous Doppler alignment and high-quality signal acquisition. Errors may arise from misalignment, suboptimal tracing, or inadequate cycle averaging. Physiological and structural conditions also influence results: moderate or severe aortic regurgitation and subaortic obstruction (fixed or dynamic) can overestimate flow. Mechanical circulatory support devices, such as Impella, can further alter native flow dynamics and waveform morphology.^1^

When technical limitations or confounding pathology exist, interpretation should rely on serial trends and integration with other haemodynamic parameters. Despite these caveats, LVOT-VTI remains a robust, versatile, and reproducible index of cardiac performance, providing a simple non-invasive approach for haemodynamic monitoring in acute cardiovascular care.

## Correlation between LUS, VExUS, and invasive haemodynamics

Elevated intracardiac filling pressures are hallmark features of ADHF. While right heart catheterization (RHC) remains the gold standard for their measurement, its invasive nature and procedural risks limit its routine use. Non-invasive echocardiographic estimation relies on complex algorithms that may be difficult to apply consistently in routine clinical practice, especially outside expert hands. In this context, LUS and VExUS have emerged as accessible bedside tools to detect elevated left and right-sided filling pressures, respectively.

In all cited invasive validation studies, left ventricular filling pressure (LVFP) was derived from pulmonary capillary wedge pressure (PCWP); therefore, ‘LVFP’ in this section refers to PCWP as the reference standard. In a prospective study of 81 patients with dyspnoea undergoing coronary angiography, the total number of B-lines was significantly higher in those with elevated LVFP ≥20 mmHg, with a median of 17 vs. 1 B-line using an 8-zone protocol. When added to clinical variables, B-line quantification improved diagnostic accuracy and net reclassification. Notably, the presence of ≥8 B-lines yielded an AUC of 0.936 (95% CI: 0.860–1.000) for predicting elevated LVFP. In contrast, the ASE/EACVI 2016 echocardiographic algorithm did not significantly improve either metric, reinforcing the value of LUS as a simple and additive tool for estimating elevated LVFP.^21^

In patients with CS, B-lines assessed by an 8-zone LUS protocol correlated strongly with LVFP. Dynamic changes in B-lines tracked variations in LVFP, supporting their use for serial monitoring. The presence of ≥3 B-lines in multiple zones identified LVFP ≥15 mmHg with good accuracy (AUC = 0.81, 95% CI: 0.72–0.89), highlighting LUS as a practical semi-quantitative tool to estimate pulmonary congestion and detect elevated LVFP in this population.^22^

Complementing this, VExUS is a reliable bedside tool for detecting elevated RAP. In a validation study comparing VExUS to invasively measured RAP, there was a clear and progressive increase in median RAP with rising VExUS grades, and grades 2–3 were typically associated with elevated RAP. For identifying RAP ≥12 mmHg, VExUS demonstrated excellent diagnostic accuracy (AUC = 0.99; 95% CI: 0.96–1.00), outperforming IVC diameter alone (AUC = 0.79; 95% CI: 0.65–0.92).^23^ The mVExUS retained good performance, reinforcing its utility as a practical bedside tool for estimating systemic venous congestion.^13^

In patients with left ventricular assist devices, PV pulsatility and the renal venous stasis index (RVSI) have been shown to discriminate between those with RAP >7 mmHg and <7 mmHg, with RVSI emerging as the most accurate variable. These Doppler-based parameters may therefore support haemodynamic assessment and management in this growing patient population.^24^

**Figure 3:**
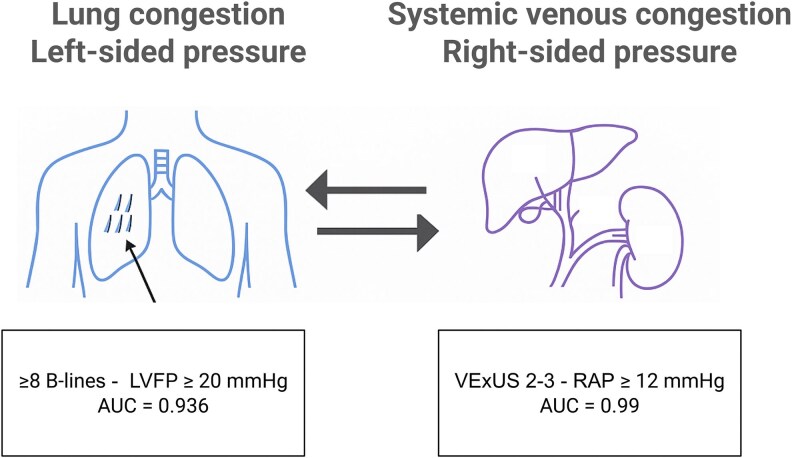
Complementary roles of LUS and VExUS in assessing haemodynamic congestion. LUS identifies pulmonary congestion and elevated left-sided filling pressures. VExUS assesses systemic venous congestion related to right-sided pressures.

Although current studies are small, single-centre and heterogeneous, they consistently show that LUS and VExUS provide complementary insights into left- and right-sided filling pressures. LUS reflects upstream pulmonary congestion, whereas VExUS captures downstream venous congestion. Together, they bridge the gap between clinical assessment and invasive haemodynamics, enhancing bedside evaluation as illustrated in *Figure 3*.

## Acute decompensated heart failure

**Figure 4:**
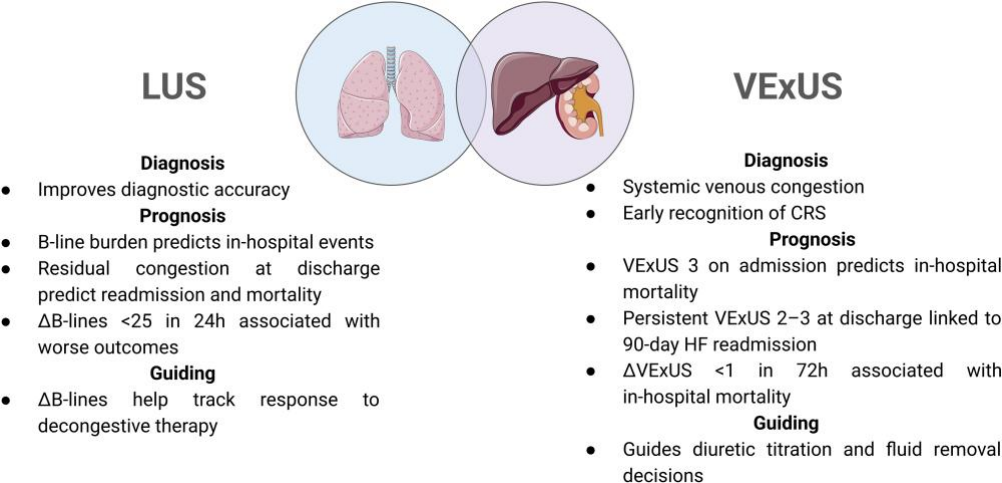
Complementary clinical roles of LUS and VExUS in ADHF. Both tools contribute to diagnosis, prognosis, and therapy guidance, offering a non-invasive and dynamic bedside approach.

The clinical utility of LUS and VExUS in HF goes beyond their haemodynamic correlations. These tools provide rapid, non-invasive insights into distinct—but complementary—aspects of haemodynamic stress: LUS reflects pulmonary interstitial fluid accumulation, while VExUS captures systemic venous congestion. Together, they inform diagnostic, prognostic, and therapeutic decision-making throughout the course of ADHF as illustrated in *Figure 4*.

### Diagnosis

LUS has emerged as a frontline tool for diagnosing ADHF, outperforming traditional modalities. In most validation studies, pulmonary congestion was defined by the presence of ≥2 B-line zones using standardized 8-zone protocols. A meta-analysis demonstrated its superior accuracy over physical examination, chest X-ray, and natriuretic peptides for confirming ADHF.^25^ In a randomized trial, the addition of LUS to clinical evaluation improved diagnostic accuracy compared to the chest X-ray/NT-proBNP approach (AUC 0.95 vs. 0.87), significantly reducing diagnostic errors (7.98 vs. 2.42 per 100 patients) and also leading to shorter time to disposition and hospital length of stay.^26,27^

VExUS complements pulmonary assessment by capturing systemic venous congestion—often the hidden driver of organ dysfunction in fluid-overloaded states. Although no studies have directly compared whether adding VExUS to standard evaluation improves diagnostic accuracy for ADHF, higher VExUS grades^2–3^ have been associated with cardiorenal syndrome, a condition frequently diagnosed in hindsight.^28^ VExUS offers a more proactive approach, detecting venous congestion even at the peak of renal injury. Its specificity and positive predictive value for identifying congestive acute kidney injury (AKI) have been consistently high.^9,29^ Notably, improvement in creatinine during decongestion parallels normalization of venous Doppler patterns and reduction in VExUS grade, reinforcing its potential utility in guiding diagnosis.^12^

### Prognostic

Pulmonary congestion assessed by LUS on admission holds independent prognostic value. In ADHF, ≥45 B-lines early during hospitalization is associated with higher mortality and readmission risk.^30^ More recently, dynamic improvement—reflected by a ≥25 B-line reduction within 24 h—has emerged as an even stronger predictor of favourable outcomes, meaning lower mortality and heart failure rehospitalization.^31^ Residual pulmonary congestion at discharge also carries powerful prognostic weight: ≥30 B-lines at discharge conferred a seven-fold increase in adverse events, and ≥3 B-lines in at least one zone per hemithorax on the 8-zone protocol independently predicted worse 100-day outcomes.^30,32,33^

Similarly, severe systemic venous congestion with VExUS grade 3 on admission has been linked to in-hospital mortality.^34^ More recent evidence suggests that dynamic changes appear even more informative: lack of improvement in mVExUS grade after 72 h (referred to as ΔVExUS) independently predicts in-hospital death.^14,35^ Finally, residual systemic congestion at discharge—particularly VExUS grade ≥2—also forecasts higher readmission rates within 90 days.^36^ Collectively, these findings highlight POCUS as a powerful tool not only for diagnosis but also for risk stratification in ADHF.

### Monitoring/therapy

In ADHF, both LUS and VExUS respond dynamically to decongestive therapy. Decreasing B-lines and normalization of venous Doppler waveforms mirror physiological improvement, though the timing and degree of change may differ across compartments.^12,33,37^ Persistently elevated values in either tool predict adverse events. Failure to reduce ≥25 B-lines within the first 24 h correlates with a higher risk of mortality and rehospitalization over a median follow-up of 217 days.^31^ Likewise, failure to achieve at least a one-grade reduction in VExUS within 72 h predicts in-hospital mortality.^35^

Beyond prognosis, both tools support therapeutic decisions. Higher VExUS grades have been linked to diuretic resistance, helping refine dosing strategies.^38^ In patients with deteriorating renal function, VExUS can clarify whether ongoing fluid removal is warranted despite clinical uncertainty regarding venous congestion.^39,40^ Thus, POCUS not only quantifies lung and systemic congestion non-invasively but also allows real-time monitoring to guide and individualize treatment.

### Integration into clinical pathways

The integration of POCUS into guideline-directed management of ADHF is increasingly feasible and supported by accumulating evidence. It should be systematically integrated as a tool for diagnosis, monitoring, and discharge readiness. At admission, combined assessment with LUS and VExUS allows rapid identification of pulmonary and systemic congestion, defining the initial haemodynamic burden and in-hospital risk. Serial reassessments within the first 24–72 h are essential to monitor therapeutic response—targeting B-line reduction on LUS and at least a one-grade improvement in VExUS, both associated with lower mortality and rehospitalization.

Daily integration of POCUS during ward rounds refines diuretic titration, ensuring dynamic adjustment of therapy according to real-time congestion status. Before discharge, a final POCUS evaluation should confirm the absence of residual congestion, as persistent pulmonary or venous overload strongly predicts early readmission.

Although the role of LVOT-VTI in this setting remains less defined as a prognostic or therapeutic target, it may complement congestion parameters by providing a non-invasive surrogate of forward flow—values <16 cm have been associated with higher mortality in cardiology intensive care units. When integrated with FoCUS, which allows rapid evaluation of ventricular function and detection of underlying triggers such as acute valvular dysfunction or wall motion abnormalities, the haemodynamic picture becomes more complete. Incorporating these parameters into serial POCUS assessments further refines bedside profiling, linking perfusion and decongestion targets within a unified physiologic framework.

Together, the structured use of POCUS modalities—primarily LUS and VExUS for assessing pulmonary and systemic congestion, and complemented by FoCUS and LVOT-VTI for evaluating ventricular function and forward flow—transforms POCUS from an ancillary test into a practical, physiology-guided pathway for precision decongestion and discharge optimization in ADHF.

## Acute myocardial infarction

### Diagnosis

In the context of AMI, the use of POCUS has recently been emphasized in the 2025 Guideline on Cardiac Ultrasound in Cardiovascular Emergency and Critical Care, endorsed by the European Association of Cardiovascular Imaging, underscoring its utility in confirming diagnosis, establishing differential diagnoses, assessing global and regional ventricular function, and detecting early complications, including mechanical complications and intraventricular thrombus formation.^5^

### Prognostic

In patients with STEMI, LUS enhances early risk stratification by detecting B-lines more sensitively than physical examination. When added to the Killip classification, LUS improved risk prediction with a net reclassification improvement of 0.18, indicating more accurate categorization of patients regarding in-hospital mortality. Notably, the absence of B-lines—‘dry lungs’—showed a 98.1% negative predictive value for in-hospital mortality.^41^ Beyond the acute phase, LUS performed within the first 24 h also predicted major adverse cardiovascular events at 1 year and enhanced the prognostic utility of the GRACE score.^42^

An integrated approach combining LUS and LVOT-VTI, referred to as the LUV classification, has been proposed to enhance early risk stratification in patients with STEMI. It allows identification of pulmonary congestion and/or low cardiac output by incorporating two key parameters: the presence of ≥3 B-line-positive lung zones and a LVOT-VTI threshold of 14 cm. Based on these criteria, patients are categorized into four haemodynamic phenotypes: A (dry lungs and LVOT-VTI >14 cm), B (wet lungs and LVOT-VTI >14 cm), C (dry lungs and LVOT-VTI ≤14 cm), and D (wet lungs and LVOT-VTI ≤14 cm). In a prospective cohort of 308 STEMI patients, in-hospital mortality ranged from 0% in LUV A to 45% in LUV D (AUC 0.915). Likewise, the incidence of acute myocardial infarction-related cardiogenic shock (AMI-CS) within the first 24 h increased progressively across categories, reaching 30.8% in LUV D. When compared to the Killip classification or protocols based solely on LUS, the LUV classification demonstrated superior performance in predicting the risk of mortality or CS during the acute phase of STEMI, resulting in a positive net-reclassification improvement of 0.07 to predict in-hospital mortality.^19,43^ Despite the promising results, validation of the LUV classification in large multicentre cohorts is essential. In addition, it is important to highlight that the use of POCUS must not delay reperfusion workflows or guideline-directed therapy.

Interestingly, in STEMI, B-lines may not exclusively reflect elevated filling pressures. A study comparing LUS to invasively measured LVFP found no significant correlation at admission, with multiple B-lines frequently present even in patients with normal LVFP, suggesting that mechanisms such as increased vascular permeability due to a proinflammatory state may underlie these findings.^44^ Nevertheless, their presence consistently correlates with worse outcomes, reinforcing the independent prognostic value of LUS in this context.

The application of VExUS in STEMI has also demonstrated clinical value. The presence of VExUS ≥1 has been associated with higher in-hospital mortality, lower cardiac index, and was more frequently observed in inferior myocardial infarctions.^45^ Additionally, a separate study reported that increasing VExUS grades were linked to a higher risk of developing AKI in patients with acute coronary syndromes, including those with non-STEMI presentations.^46^

### Integration into clinical pathways

**Figure 5:**
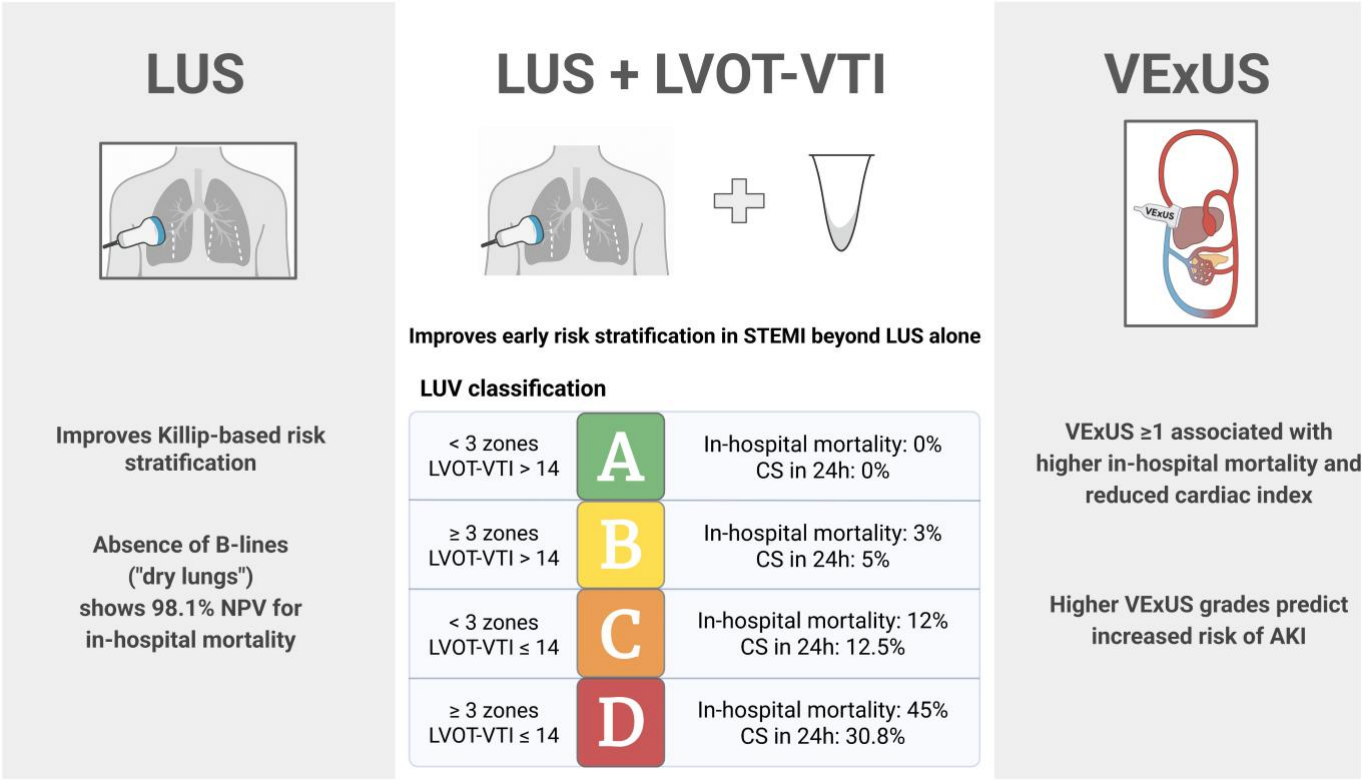
Prognostic role of POCUS in STEMI. **LUS:** improves Killip-based risk stratification. **LUS**  **+**  **LVOT-VTI (LUV Classification):** combines lung congestion and low cardiac output to stratify risk of in-hospital mortality and evolution to CS across groups. **VExUS:** VExUS grade ≥1 is associated with higher in-hospital mortality, lower cardiac index, and increased risk of AKI.

The integration of POCUS into the clinical management of AMI represents a significant advancement in bedside cardiovascular assessment. Beyond confirming left ventricular systolic dysfunction, POCUS enables rapid regional wall motion analysis, allowing early recognition of segmental abnormalities that support the diagnosis and localization of infarction. Furthermore, it facilitates prompt detection of mechanical complications and helps rule out alternative diagnoses that may mimic AMI, including acute aortic syndromes or massive pulmonary embolism. The addition of LUS and LVOT-VTI assessment extends its role from diagnosis to prognostication, providing non-invasive insights into pulmonary congestion, haemodynamic status, and cardiac output, as shown in *Figure 5*. When systematically incorporated into early clinical pathways, POCUS serves as a complementary tool that refines diagnostic accuracy, risk stratification, and therapeutic decision-making in patients presenting with acute coronary syndromes.

## Cardiogenic shock

### Haemodynamic profiling

In experienced hands, POCUS serves as a powerful tool for echodynamic (non-invasive haemodynamic monitoring) and early differentiation of shock subtypes. A recent meta-analysis demonstrated a pooled sensitivity of 90% and specificity of 98% for identifying the underlying shock mechanism—including a specificity of 98% (95% CI: 97–99) for CS—highlighting its strong diagnostic performance compared with invasive reference standards.^47^

Beyond initial classification of shock type, echodynamic can approximate key haemodynamic parameters in ADHF-related cardiogenic shock (ADHF-CS). Compared with invasive measurements, echocardiographic cardiac index (eCI ≤2.2 L/min/m²) showed high sensitivity (97%) and acceptable specificity (73%) for detecting low-output states. Estimates of pulmonary artery pressures, RAP, cardiac power output (eCPO), and Pulmonary Artery Pulsatility Index (ePAPi) also show good diagnostic performance, although accuracy is lower for the estimation of PCWP. Notably, a reduced ePAPi was associated with higher 60-day mortality, while lower eCPO showed a similar trend. Echocardiographic phenotyping according to the Tehrani classification^48^—distinguishing predominant left, right, or biventricular dysfunction—correlated well with invasive haemodynamic profiles (κ = 0.457; *P* < 0.001).^49^

In addition, LVOT-VTI provides a practical, non-invasive surrogate of forward flow that can be used to monitor cardiac output trends and assess response to therapy. A markedly reduced VTI (<10 cm) has also been proposed as a threshold indicating severe pump failure and insufficient native output in patients supported by mechanical circulatory devices such as VA-ECMO, while progressive improvement with VTI >10 cm may signal recovery of systolic function and readiness for weaning. Nonetheless, structural and functional findings must always be interpreted in conjunction, ensuring that VTI trends are contextualized within the broader echocardiographic assessment.^17,50^

**Figure 6:**
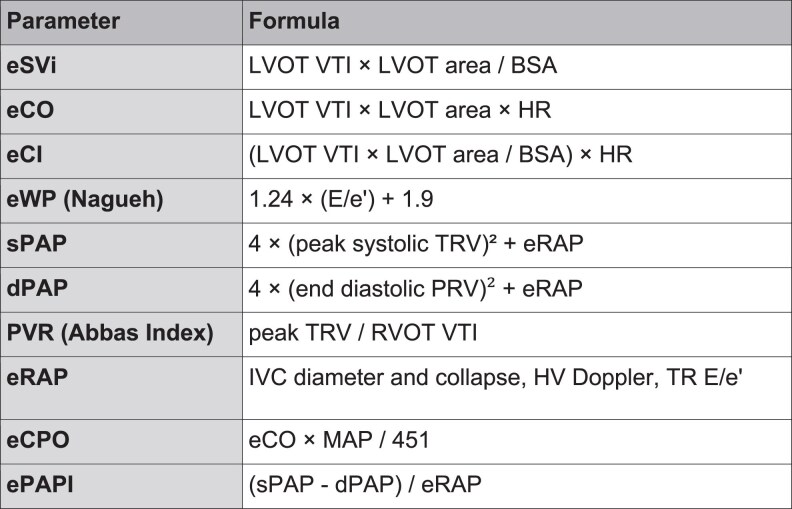
Formulas for estimated haemodynamic parameters derived from echocardiography. Abbreviations: eSVI: estimated stroke volume index; eCO: estimated cardiac output; eCI: estimated cardiac index; eWP: estimated wedge pressure (Nagueh’s formula); sPAP: systolic pulmonary artery pressure; dPAP: diastolic pulmonary artery pressure; PVR: pulmonary vascular resistance; eRAP: estimated right atrial pressure; eCPO: estimated cardiac power output; ePAPI: estimated pulmonary artery pulsatility index. Other variables: LVOT: left ventricular outflow tract; VTI: velocity time integral; BSA: body surface area; TRV: tricuspid regurgitation velocity; TR: tricuspid regurgitation; PRV: pulmonary regurgitation velocity; MAP: mean arterial pressure; SBP/DBP: systolic/diastolic blood pressure; E/e′: ratio of early mitral inflow velocity to early diastolic mitral annular velocity; TAPSE: tricuspid annular plane systolic excursion.

These findings support the role of serial, non-invasive echocardiographic assessment to monitor haemodynamic response and guide therapy at the bedside. Nevertheless, RHC remains the reference standard for precise quantification of intracardiac pressures and cardiac output. Key echocardiographic formulas for these parameters are summarized in *Figure 6*.

### Risk stratification beyond SCAI

LUS provides prognostic value in CS by detecting and tracking pulmonary congestion. In the ALTShock-2 registry, a simplified 4-zone LUS protocol using a dichotomous threshold (≤50% vs. >50% B-line involvement) was applied at admission and 24 h in patients with CS. Patients with >50% B-lines at 24 h had significantly higher 30-day mortality, while early B-line reduction was independently associated with improved survival.^51^

Similarly, in a prospective study of STEMI patients in AMI-CS, pulmonary congestion assessed by LUS was strongly associated with SCAI shock stage—each stage increment corresponded to a 2.2-fold increase in the odds of having more B-line–positive lung zones. LUS also enabled meaningful risk reclassification: the presence of pulmonary congestion (‘wet lungs’, defined as ≥3 B-lines in ≥2 lung zones) identified patients at higher risk of in-hospital mortality, even among those initially categorized as low-risk (SCAI A or B). Among SCAI A/B patients, those with wet lungs had substantially higher mortality than those with dry lungs (8.8% vs. 2.7%; adjusted OR 3.5, 95% CI 1.4–8.6; *P* = 0.006), underscoring the additive prognostic value of LUS.^52^ Together, these findings underscore the value of serial LUS for dynamic risk stratification and bedside phenotyping in CS.

### Refining diagnosis beyond haemodynamics and post-procedural complications

Beyond haemodynamic profiling, bedside echocardiography with FoCUS extends the diagnostic scope by allowing rapid structural evaluation of the heart—an advantage over purely catheter-based monitoring. In AMI-CS, FoCUS enables the detection of mechanical complications such as ventricular septal defect, papillary muscle rupture, or free wall rupture. In ADHF-CS, it facilitates recognition of contributory or secondary causes, including acute valvular dysfunction or pulmonary embolism.^5^

FoCUS also plays a critical role in identifying obstructive forms of shock caused by cardiac tamponade, acute right ventricular afterload increase, or dynamic LVOT obstruction.^36^

Finally, in patients developing shock after cardiac or structural interventions, FoCUS is particularly valuable for the prompt detection of pericardial effusion and tamponade—conditions often missed by physical examination, ECG, or chest radiography. In a retrospective study, FoCUS significantly shortened both the time to diagnosis and the time to pericardiocentesis compared with standard imaging, underscoring its life-saving potential in this critical setting.^53,54^

### Integration into clinical pathways

POCUS provides a comprehensive, non-invasive framework for the haemodynamic evaluation of CS—supporting both initial profiling and dynamic management after therapeutic interventions. At presentation, it helps discriminate predominant mechanisms such as pump failure, distributive physiology, or volume depletion, thereby guiding early treatment choices. Once the phenotype is established, serial echocardiographic assessment allows real-time estimation of filling pressures, pulmonary artery pressures, cardiac output, ventricular function, and vascular resistance to titrate vasoactive therapy. These measurements should be repeated after major haemodynamic shifts or medication adjustments. Their reliability depends on adequate acoustic windows and operator expertise; when image quality is limited, or Doppler data are inconsistent, RHC remains the reference standard.

FoCUS should be integrated at the initial assessment to aid in the structural evaluation of the heart, helping to differentiate shock phenotypes and identify mechanical complications, significant valvular disease, or pericardial effusion that may alter management.

LUS complements this approach by refining risk stratification within the SCAI framework. In patients classified as SCAI stages A or B, the presence of ‘wet lungs’ was associated with significantly higher in-hospital mortality compared with ‘dry lungs’. This highlights the prognostic value of LUS even in early stages of shock, supporting its incorporation into bedside risk reclassification.

Integrating LUS with VExUS further personalizes volume management. A net negative fluid balance should be pursued, even with borderline creatinine, when venous Doppler demonstrates severe pressure transmission (VExUS grade 2–3) accompanied by pulmonary congestion (>2 B-line–positive zones).^55^ Conversely, in patients with fluid tolerance—minimal pulmonary congestion (<2 B-line—positive zones) and low systemic venous congestion (VExUS <2)—cautious fluid administration may be considered if preload responsiveness is demonstrated (LVOT-VTI increase >15% during passive leg raising).

Through this integration, haemodynamic echocardiography bridges diagnostic assessment and real-time therapeutic guidance, aligning non-invasive monitoring with established critical care frameworks and facilitating individualized management in CS.

## Implementation

Effective integration of POCUS into acute cardiovascular care requires structured education, certification, and standardized protocols. Learning curves vary across modalities: LUS typically requires fewer supervised scans, while ∼50 supervised examinations appear sufficient to achieve proficiency in VExUS interpretation. More advanced haemodynamic measurements demand extended supervised training until reproducibility approaches that of experienced echocardiographers.^15,56^ Importantly, targeted training of cardiology residents has already proven effective in improving clinical decision-making—particularly for the recognition of mechanical complications in acute myocardial infarction.^57^

Defining these learning curves more precisely will be essential to ensure safe and effective implementation. Standardized acquisition protocols and evidence-based guidance on appropriate indications should be widely disseminated, initially focusing on emergency physicians, intensivists, and cardiologists, to promote the integration of POCUS into daily clinical workflows.

## Limitations

Despite its rapid adoption, POCUS remains highly operator-dependent and subject to variability in image quality, interpretation, and documentation. Technical limitations—such as poor acoustic windows, motion artefacts, and Doppler misalignment—can compromise accuracy. The pitfalls of each method are described in detail in the preceding topics and must always be considered in conjunction with the patient's clinical presentation. Additionally, the lack of standardized training pathways and certification requirements across institutions hinders reproducibility and broad clinical adoption.

Moreover, evidence directly linking POCUS-guided strategies to improved patient-centred outcomes remains limited. Most studies are single-centre and observational, emphasizing the need for larger, pragmatic trials to clarify its incremental value over conventional assessment and to establish its role in guideline-directed management.

## Future directions

Beyond implementation and standardization, evidence generation and technological innovation will define the next phase of POCUS evolution. Multicentre randomized trials should evaluate the impact of POCUS-guided strategies on hard outcomes—such as mortality, rehospitalization, and cost-effectiveness—while pragmatic implementation studies in emergency departments, intensive care units, and cardiology wards can inform real-world integration. Establishing competency frameworks and quality assurance processes will be essential to ensure consistent performance.

In parallel, artificial intelligence (AI) is expected to play an increasingly supportive role in POCUS by reducing inter-operator variability, guiding less-experienced users during image acquisition, and assisting with automated recognition of key sonographic patterns. Deep-learning models already demonstrate the ability to standardize echocardiographic acquisition and interpretation, and these principles are directly applicable to POCUS modalities such as LUS and VExUS, where automated B-line quantification and waveform pattern recognition could enhance reproducibility. In the future, AI may also integrate ultrasound findings with clinical and laboratory data to generate real-time haemodynamic profiles, improving diagnostic accuracy and prognostic stratification in acute cardiovascular care.^58,59^ Collectively, these developments will consolidate POCUS as a widely applicable, high-impact tool in cardiovascular care—bridging physiology, technology, and clinical decision-making—while emphasizing its complementarity, rather than superiority, to traditional diagnostic tools except where evidence clearly supports it.

## Conclusion

The use of POCUS represents one of the major recent revolutions in cardiovascular care and in modern cardiopulmonary diagnostics. Its immediate availability at the bedside empowers clinicians to expand diagnostic capabilities, accelerate decision-making, and provide more precise risk stratification—often in real time and at the point of greatest need.

As evidence continues to accumulate, POCUS is increasingly recognized not as a replacement for traditional diagnostic tools, but as their most powerful complement—integrating clinical reasoning with physiologic imaging to refine assessment and guide therapy. Its thoughtful incorporation into daily practice is both feasible and essential to deliver more timely, individualized, and efficient care in the era of precision medicine.

## Data Availability

No new data were generated or analysed in support of this research.

## Lead author biography

Dr Marina Saadi completed residencies in Internal Medicine, Cardiology, and Echocardiography at Hospital de Clínicas de Porto Alegre, Brazil. She recently completed a fellowship in cardiovascular imaging and structural heart interventions at the Core Lab of CHU Rennes, France, under the mentorship of Professor Erwan Donal. In Brazil, she also works in the Cardiac Intensive Care Unit, with a focus on integrating advanced imaging into clinical decision-making for critically ill cardiac patients. Her academic work centers on point-of-care ultrasound and venous congestion assessment. She completed her PhD on the prognostic value of the VExUS score in acute decompensated heart failure and is dedicated to advancing and disseminating knowledge in this field.
